# Characterization of a novel Carbon Dot Inhibitor of Tau and Amyloid Beta Aggregation in Alzheimer’s Disease

**DOI:** 10.1002/alz.093558

**Published:** 2025-01-09

**Authors:** Hannah J Burr, Nathan Scott Smith, Wei Zhang, Chunyu Wang

**Affiliations:** ^1^ Rensselaer Polytechnic Institute, Troy, NY USA; ^2^ University of Miami, Miami, FL USA

## Abstract

**Background:**

Alzheimer’s Disease (AD) is a neurodegenerative disorder whose pathological hallmarks include tau and amyloid beta aggregation, a phenomenon that has been linked to inflammation and degradation of brain tissue. Prior data published in the Wang lab suggests that carbon dots (CDs) synthesized from citric acid and urea can inhibit aggregation. We sought to characterize the inhibitory effects of a new class of CDs synthesized from varied ratios of Congo red and citric acid. Since Congo red carbon dots (CRCDs) can cross the blood‐brain barrier (BBB), they have the potential to be used as a therapeutic.

**Method:**

A new type of CD was synthesized via the muffle furnace method using citric acid, urea, thiourea, selenourea, formamide, and congo red. The CDs have a diameter less than 20 nanometers, enabling them to cross both the BBB and the cell membrane, which was confirmed experimentally with zebrafish models. The newly synthesized CDs were used in Thioflavin T (ThT) aggregation assays to assess protein aggregation inhibition. This was verified visually by Atomic Force Microscopy (AFM).

**Result:**

All three CRCDs inhibited tau aggregation, with CRCD1 being the most effective. CRCD1 had the lowest IC50 of 0.21ug/mL, while CRCD2 and CRCD3 had an IC50 of 0.50ug/mL and 2.55ug/mL, respectively. All three CRCDs also inhibited amyloid beta aggregation, with CRCD being the most effective. CRCD1 had the lowest IC50 of 1.76 ug/mL, while CRCD2 and CRCD3 had an IC50 of 3.34 ug/mL and 5.61 ug/mL, respectively.

**Conclusion:**

The ability to reduce tau aggregation in the brain has the potential to slow the growth of the disease and potentially alleviate symptoms. There are currently limited treatments available for AD, but CRCDs show great promise.

